# Consideration of Age Is Necessary for Increasing the Accuracy of the AJCC TNM Staging System of Pancreatic Neuroendocrine Tumors

**DOI:** 10.3389/fonc.2019.00906

**Published:** 2019-09-19

**Authors:** Zhengshi Wang, Wenli Jiang, Lijuan Zheng, Jie Yan, Jiaqi Dai, Caiguo Huang, Qian Zhang, Zhiqiang Yin, Xiangnan Gong, Yun Zhang

**Affiliations:** ^1^Thyroid Center, Shanghai Tenth People's Hospital, Tongji University School of Medicine, Shanghai, China; ^2^Shanghai Center for Thyroid Diseases, Shanghai, China; ^3^Department of Biochemistry and Molecular Biology, The Faculty of Basic Medical Science, Second Military Medical University, Shanghai, China; ^4^Department of Gastroenterology, Gansu Provincial Hospital, Lanzhou, China; ^5^Department of Nuclear Medicine, Shanghai Tenth People's Hospital, Tongji University School of Medicine, Shanghai, China; ^6^Department of Thoracic Surgery, Xuzhou Central Hospital, The Affiliated Xuzhou Hospital of Medical College of Southeast University, Xuzhou, China

**Keywords:** pancreatic neuroendocrine tumor, AJCC, overall survival, SEER, age

## Abstract

**Purpose:** Currently, of the two most common staging systems of pancreatic neuroendocrine tumors (pNETs) one is from the European Neuroendocrine Tumor Society (ENETS) and the other is from the American Joint Committee on Cancer (AJCC). However, there are imperfections in both these staging systems.

**Patients and methods:** Patients were selected retrospectively from the Surveillance Epidemiology and End Results (SEER) database (2004 to 2013). The effect of age on the hazard ratio (HR) was evaluated using restricted cubic splines. The discriminatory power of the staging systems was determined using the concordance index (C-index).

**Results:** A total of 3,034 patients with pNETs were included in the final analyses. The risk of death increased slowly along with age for patients under 60 years of age, but the risk of death rose sharply for those over 60 years of age, forming a mirrored L-shaped survival curve. In the current AJCC tumor-node-metastasis (TNM) staging system, no statistical significance was observed between stages IA and IB (*p* = 0.105). Patients with stage IIB even had longer OS than patients with IIA, although there was no statistical significance (*p* = 0.574). The proportion of stage III patients was small (2.7%). In the proposed aTNM staging system, significant survival differences could be observed among stage I, IIA, and IIB (*p* < 0.001) and the proportion of stage III rose from 2.7 to 25.7%.

**Conclusion:** Our findings suggest that age has a critical influence on the survival of patients with pNETs. Age should be considered as a factor in future staging systems of pNETs.

## Introduction

Pancreatic neuroendocrine tumors (pNETs) are rare, with an incidence rate of >0.5/100,000 in 2004 in the United States (1), and represent ~1–2% of all pancreatic neoplasms (2). Most pNETs are sporadic and may also occur with some inherited genetic syndromes, the most two common of which were multiple endocrine neoplasia (MEN) types 1 and MEN2. The former are related to the mutated menin gene and the latter are associated with the mutated RET gene. pNETs are usually indolent and have a more favorable outcome (3). However, factors such as the diagnosis, treatment and prognosis of pNETs have not been clearly understood compared with other pancreatic exocrine tumors. Therefore, a well-defined, useful classification is needed to better manage patients with pNETs.

pNETs can be classified into different groups depending on different characteristics. According to the molecular mutations, pNETs are divided into sporadic pNETs and hereditary pNETs. According to the functionality, pNETs are distinguished in functional and non-functional. Among the functional pNETs, insulinomas account for 70% approximately and 15% are glucagonomas. Other rare functional pNETs include pancreatic polypeptidoma (PPoma), VIPomas secreting vasoactive intestinal polypeptide (VIP) and cholecystokininoma (CCKoma). According to the histological types, pNETs are divided into Grade 1, Grade 2 and Grade 3 based on ki-67 index, mitotic count, and proliferative activity of tumor cells (4). To guide clinical practice, of the two most common staging systems for pNETs, one is from the European Neuroendocrine Tumor Society (ENETS) and the other is from the American Joint Committee on Cancer (AJCC). However, through the ENETS staging system a similar outcome is observed for stage I and stage IIA (5, 6). Luo et al. (7) attempted to modify the ENETS (mENETS) staging system by adopting AJCC staging definitions and ENETS T, N, and M definitions. Unfortunately, in the mENETS staging system patients with stage IA still had a similar prognosis to those with stage IB. In the AJCC staging system, only a small percentage of patients, ranging from 4 to 5.3%, had a stage III prognosis (8, 9). Even in the mENETS staging system, stage III prognosis accounted for merely 8.9% of patients (7). These observations suggest that current staging systems (AJCC, ENETS and mENETS) still need improvement.

Multiple studies have shown that the main prognostic factors of pNETs include tumor stage, histological grade (6), age (10, 11), functional status (11), surgical margin (12), and metastatic pattern (e.g., diffuse liver metastases, extrahepatic metastases, bone metastases) (13–16). Halfdanarson et al. (11) divided patients with pNETs into 4 groups based on age: 18–50, 51–60, 61–70, and 71–95 years. Their median overall survival (OS) was 52, 44, 19, 9.5 months, respectively. Therefore, we hypothesized that the older patients got, the worse their prognosis was. Additionally, we noticed that age was included in the AJCC tumor-node-metastasis (TNM) staging system of papillary thyroid cancer (PTC) because of the observation of a dramatic increase in the risk of death beginning around the age interval of 50–60 years (17). Thus, this study aims to validate our hypothesis and explore whether age has an influence on the AJCC TNM classification of pNETs similar to the influence it had on PTC, using the Surveillance Epidemiology and End Results (SEER) database.

## Materials and Methods

### Ethics Statement

This study was approved by the institutional review board of Shanghai Tenth People's Hospital, Tongji University School of Medicine. Patients from the Surveillance, Epidemiology, and End Results (SEER) database had previously consented to participate in any scientific research worldwide.

### Patients

The SEER database (2004 to 2013) was used to identify pNETs patients. We defined pNETs to include the following International Classification of Diseases for Oncology third edition (ICD-O3) codes: islet cell carcinoma (8150), insulinoma (8151), glucagonoma (8152), gastrinoma (8153), vipoma (8155), somatostatinoma (8156), enteroglucagonoma (8157), carcinoid (8240), enterochromaffin cell carcinoid (8241), enterochromaffin-like cell tumors (8242), goblet cell carcinoid (8243), composite carcinoid (8244), adenocarcinoid (8245), neuroendocrine carcinoma (8246), and atypical carcinoid (8249) (18). TNM classifications based on two codes: the derived AJCC stage group (6th edition; 2004+) and the derived AJCC stage group (7th edition; 2010+) were retrieved. Study enrollment criteria were patients who had a positive histological diagnosis of pNETs, definite TNM information and patients who had survival data available. The study cohort didn't distinguish radical or palliative patients. Patients were excluded if there were coexisting pancreatic adenocarcinoma. Clinical and pathological features included age, sex, race, functional status, location of the primary tumor, grade, and TNM stage.

### Statistical Analysis

Multivariate analysis, including sex, age, tumor type, stage, grade, tumor location and race, was done using the Cox proportional hazard regression model and log-rank tests were used to evaluate prognostic factors. The stepwise backward procedure based on the likelihood ratio was used in the Cox model. The hazard ratio (HR) and a 95% confidence interval (CI) were calculated. The effect of age on the HRs was evaluated using restricted cubic splines in R i386 3.3.2 software (running rms package), in which age was regarded as continuous variable. Survival analysis was conducted using Kaplan-Meier methods in PASW Statistics 18 software to assess the prognosis of pNETs patients with different stages. The concordance index (C-index) was used to determine the discriminatory ability of the staging systems. Statistical significance was defined as a two-sided *p* < 0.05.

## Results

### Patient Characteristics

Clinical and pathological features of patients enrolled in the study are listed in Table 1. A total of 3,034 patients with pNETs were included and comprised of 1,655 males and 1,379 females. The median age of all patients was 59 years (ranging from 11 to 97 years). Primary tumors were most frequently located at the body-tail of the pancreas (*n* = 1,350, 44.5%), followed by the head (*n* = 907, 29.9%). The primary tumor site, mainly included the pancreatic duct and islets of Langerhans while other specific parts of the pancreas were defined as others (*n* = 777, 25.6%). Among the available data, 1,547 cases were described on pathologic reports as well-differentiated (Grade I) or moderately differentiated (Grade II) (I-II−52.3%), and 281 were poorly differentiated (Grade III) or undifferentiated (Grade IV) (III-IV−9.3%). Apart from patients with unknown grade information, Grade I-II accounted for a majority (85.0%, 1588/1869). According to the 7th AJCC staging system, the frequency, and proportion of stage I, II, III, IV was 879 (29.0%), 607 (20.0%), 83 (2.7%), and 1,465 (48.3%), respectively.

**Table 1:** Clinical and pathological features of 3,034 patients with pNETs in the SEER database.

**Variables**	**SEER (*N* = 3,034)**	**%**
**Age, years**		
≤60	1,621	53.4
>60	1,413	46.6
Median age (range)	59 (11–97)	
**Sex**		
Male	1,655	54.5
Female	1,379	45.5
**Grade**		
I-II	1,588	52.3
III-IV	2,81	9.3
Unknown	1,165	38.4
**Tumor type**		
Functional	547	18.0
Non-functional	2,487	82.0
**Race**		
White	2,359	77.8
Black	386	12.7
Others	289	9.5
**Location**		
Head	907	29.9
Body-tail	1,350	44.5
Others	777	25.6
**AJCC stage**		
I	879	29.0
IA	444	14.6
IB	435	14.4
II	607	20.0
IIA	208	6.9
IIB	399	13.1
III	83	2.7
IV	1,465	48.3

*SEER, Surveillance Epidemiology and End Results; AJCC, American Joint Committee on Cancer*.

### Prognostic Factors of Survival

Multivariate analysis demonstrated that among factors being considered age (≤60 vs. >60), grade (I-II vs. III-IV), functional status (functional type vs. non-functional type), race (white vs. black), location (head vs. body-tail), and stage (Table 2) were independently associated with survival.

**Table 2:** Prognostic significance for OS by multivariate analysis of variables for patients with pNETs using the Cox proportional hazard regression model.

**Variables**	**HR (95% CI)**	***P***
**Age, years**		
≤60	1	
>60	1.753 (1.552–1.980)	<0.001
**Sex**		
Male	1	
Female	0.932 (0.826–1.052)	0.255
**Race**		
White	1	
Black	1.267 (1.062–1.511)	0.008
Others	0.852 (0.681–1.066)	0.161
**Grade**		
I-II	1	
III-IV	4.427 (3.635–5.392)	<0.001
Unknown	2.378 (2.036–2.778)	<0.001
**Tumor type**		
Functional	1	
Non-functional	1.632 (1.240–2.147)	<0.001
**Location**		
Head	1	
Body-tail	0.834 (0.721–0.964)	0.014
Others	0.975 (0.838–1.135)	0.746
**AJCC stage**		
IA	1	
IB	1.589 (0.908–2.781)	0.105
IIA	2.687 (1.528–4.723)	0.001
IIB	2.481 (1.471–4.187)	0.001
III	5.969 (3.373–10.563)	<0.001
IV	9.601 (5.984–15.404)	<0.001

*CI, confidence interval; HR, hazard ratio*.

### Effect of Age on Staging

**Figure 1:**
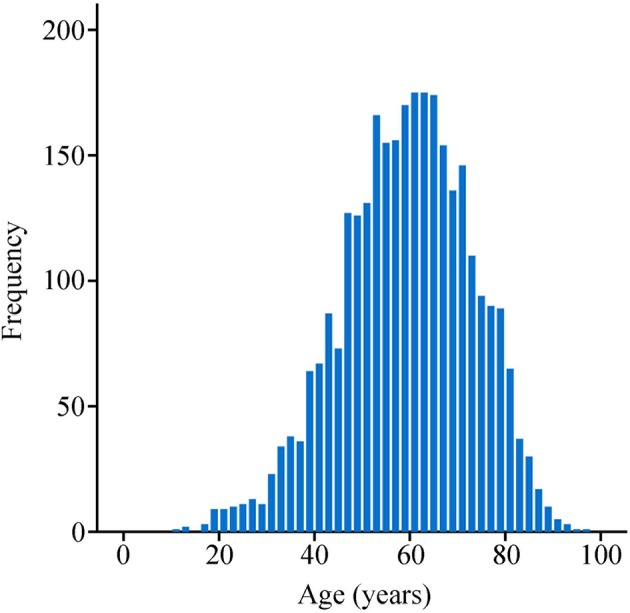
Age distribution of entire cohort.

**Figure 2:**
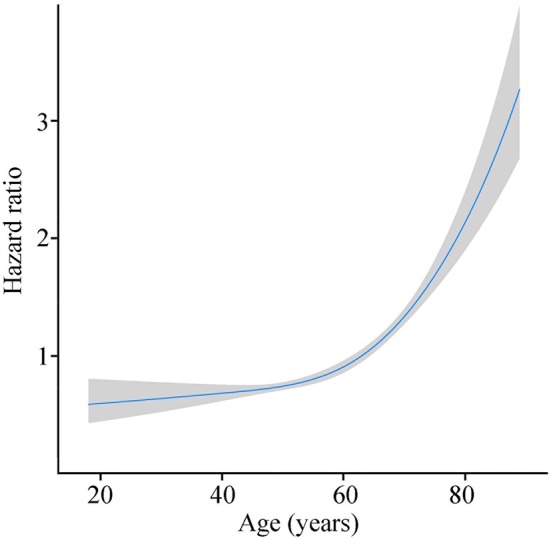
Risk of death with age growing by R software (blue line stands for HR and gray area for 95% CI).

**Figure 3:**
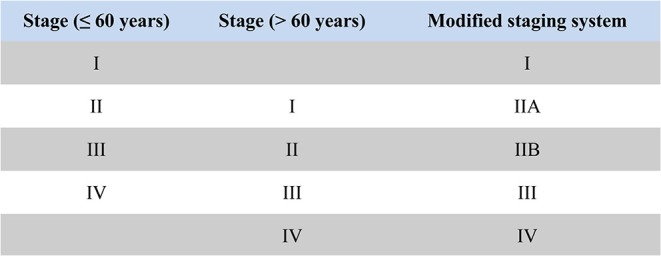
The principle diagram of modifying AJCC TNM staging system based on age: stage I patients under 60 years were classified as stage I in the modified staging system; stage II patients under 60 years and stage I patients older than 60 years were classified as stage IIA in the modified staging system; stage III patients under 60 years and stage II patients older than 60 years were classified as stage IIB in the modified staging system; stage IV patients under 60 years and stage III patients older than 60 years were classified as stage III in the modified staging system; stage IV patients older than 60 years were classified as stage IV in the modified staging system.

Mean age of patients was 58.7 years (interquartile range, 50 to 69 years, Figure 1), and approximately equaled median age. In a univariable Cox model for OS, the effect of age was shown by a mirrored L-shaped curve (Figure 2), the first half steadily increasing and the latter half showing a sharp increase. The turning point was approximately around the age of 60 years. As shown in Table 2, the hazard ratio (HR) of death for patients over 60 years was 1.753 in reference to patients under 60 (*p* < 0.001). Notably, the oldest group (>75 years) showed an increase in the risk of death as high as 235.6% over that of the youngest group (<40 years) (HR- 3.356; 95% CI−2.544–4.426, *p* < 0.001). Thus, we included age as a factor in the TNM staging system. The principle of modifying the TNM staging system was that patients over 60 years of age were upstaged as the next advanced stage (Figure 3), resulting in modified stages of I, IIA, IIB, III, and IV. To increase the discriminatory ability, stage IA and stage IB were classified as stage I. Supplementary Figure 1 provided another more detailed version of modified stages in which stage IA, stage IB, stage IIA, and stage IIB were regarded as an individual group, respectively.

**Figure 4:**
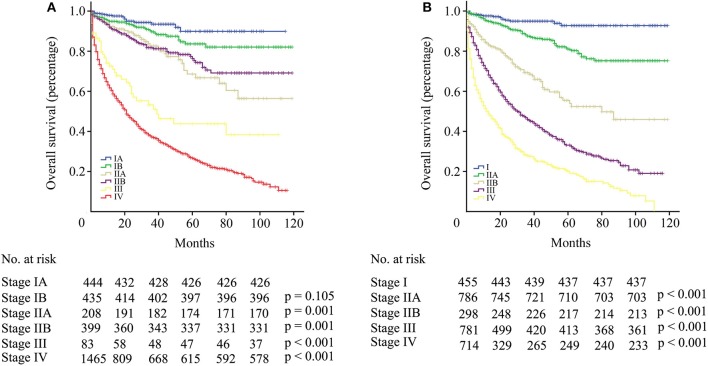
**(A)** Survival curve of patients with pNETs according to the AJCC TNM staging system. **(B)** Survival curve of patients with pNETs according to the aTNM staging system.

The difference in survival between each stage was assessed according to the AJCC TNM staging system. Between stage IA and IB, no statistical significance was observed (*p* = 0.105, Figure 4A). Patients with stage IIB even had longer OS than patients with IIA, although there was no statistical significance (*p* = 0.574, Figure 4A). Significant differences were observed among stage II, III, and IV patients (*p* < 0.001), but the survival gap between stage II and III was huge (Figure 4A). According to the modified TNM staging system that included age (aTNM), a significant difference could be observed among all stages (*p* < 0.001, Figure 4B). Survival gaps between neighboring stages were generally uniformly distributed. The C-index of the TNM staging system was 0.720 (95% CI−0.685–0.755) while the C-index of the aTNM staging system was 0.753 (95% CI−0.718–0.788). Additionally, the proportion of stage III patients rose from 2.7 to 25.7% (Table 3).

**Table 3:** Comparison of pNET patient distributions between TNM and aTNM staging system by each stage.

**Stage**	**TNM (%)**	**aTNM (%)**
I	879 (29.0)	455 (15.0)
IA	444 (14.6)	-
IB	435 (14.4)	-
II	607 (20.0)	1,084 (35.7)
IIA	208 (6.9)	786 (25.9)
IIB	399 (13.1)	298 (9.8)
III	83 (2.7)	781 (25.7)
IV	1,465 (48.3)	714 (23.5)

## Discussion

pNETs are neoplasms that arise from hormone producing pancreatic islet cells (19). Until now, the influence of age on pNETs has remained to be further elucidated. In Halfdanarson's report (11), age was defined as categorical variable rather than continuous variable, which cannot fully reflect the true effect of age on the survival. This is the first study regarding age as continuous variable to investigate the effect of age on the prognosis of pNETs. The results of this study suggest that age is an independent prognostic factor of pNETs. The effect of age on the survival of patients with pNETs was specific showing a mirrored L-shaped curve. The risk of death increased slowly along with age for patients under 60 years of age. However, for patients over 60 years of age the risk of death rose sharply. Our findings indicate that patients over 60 years are a different population from those under 60 years of age. Thus, it was necessary to incorporate age into the staging system. The clinical guidelines recommended that patinets with non-functional pNETs <1 cm could be safely followed (20), which might be questioned since older patients (>60 years) showed a higher death risk. Additionally, older patients should undergo more frequent surveillance than younger patients.

Bilimoria et al. (21) first introduced the AJCC staging system for pancreatic adenocarcinoma into the staging for pNETs. However, it couldn't stratify patients with pNETs by death risk as properly as it did among patients with pancreatic adenocarcinoma. In the AJCC staging system for pNETs, the first two stages (stage IA and IB) had similar survival outcomes. When taking age into consideration, significant survival differences could be observed between the first two stages of the aTNM staging system (with stage I as the reference: HR for stage IIA−2.591, *p* < 0.001). In the AJCC staging system, there were a relatively low proportion of patients with stage III, but with the aTNM staging system, the proportion of stage III rose from 2.7 to 25.7%. Stage III patients with distant metastasis under 60 years that caused this increase were classified as stage IV in the TNM staging system.

Some studies (22–24) have argued that age has no significant impact on the survival of pNET patients, which has mainly resulted from studies with small sample sizes. In studies with large sample sizes or multicenter data, advanced age was closely related with shorter survival time (11, 25). The mechanism of how advanced age increases mortality is not yet clear. The mTOR pathway has been reported to play a critical role in senescence and senescence-related diseases (26, 27) and was frequently altered in pNETs (28). Everolimus, an mTOR inhibitor, has been proven effective in the treatment of pNETs in clinical practice (29). These findings suggest that the mTOR pathway may be the potential mechanism.

There were some limitations in this study. First, our results were based on retrospective data. Second, external data are needed to validate current conclusions. Third, a lack of genetic information hampered further exploration of the mechanisms underlying the influence of age on the prognosis of patients with pNETs. Fourth, progression-free survival data were needed to further evaluate the true effect of age on disease recurrence. Therefore, more prospective data and basic studies are needed to investigate in depth the effect of age on pNETs.

## Data Availability

Publicly available datasets were analyzed in this study. This data can be found here: https://seer.cancer.gov/.

## Ethics Statement

The studies involving human participants were reviewed and approved by the institutional review board of Shanghai Tenth People's Hospital, Tongji University School of Medicine. The patients/participants provided their written informed consent to participate in this study. Written informed consent was obtained from the individual(s) for the publication of any potentially identifiable images or data included in this article.

## Author Contributions

ZW and YZ made substantial contributions to the design of the study, carried out the analysis, interpreted the data. WJ and LZ contributed to the review of previous literature. JY and JD contributed substantially to the data discussion and critically commented on the manuscript for scientific content. All authors made substantial contributions to the conception and design of the study, data interpretation and drafting of the manuscript, were responsible for the quality of the overall manuscript. All authors approved the final version of the manuscript.

### Conflict of Interest Statement

The authors declare that the research was conducted in the absence of any commercial or financial relationships that could be construed as a potential conflict of interest.

## Supplementary Material

Supplementary Figure S1 Survival curve of patients with pNETs according to the aTNM staging system, in which stage IA, stage IB, stage IIA, and stage IIB were regarded as an individual group respectively.
